# Comparison between available early antiviral treatments in outpatients with SARS-CoV-2 infection: a real-life study

**DOI:** 10.1186/s12879-023-08538-9

**Published:** 2023-10-02

**Authors:** Matteo Rinaldi, Caterina Campoli, Mena Gallo, Domenico Marzolla, Alberto Zuppiroli, Riccardo Riccardi, Martina Casarini, Daniele Riccucci, Marta Malosso, Cecilia Bonazzetti, Renato Pascale, Beatrice Tazza, Zeno Pasquini, Lorenzo Marconi, Stefania Curti, Maddalena Giannella, Pierluigi Viale

**Affiliations:** 1 Infectious Diseases Unit, Department for Integrated Infectious Risk Management, IRCCS Azienda Ospedaliero-Universitaria di Bologna, Via Massarenti 11, Bologna, 40137 Italy; 2 Department of Medical and Surgical Sciences, Alma Mater Studiorum University of Bologna, Bologna, Italy

**Keywords:** SARS-CoV-2, Early antiviral treatment, real-life cohort

## Abstract

**Purpose:**

To investigate the clinical impact of three available antivirals for early COVID-19 treatment in a large real-life cohort.

**Methods:**

Between January and October 2022 all outpatients tested positive for SARS-CoV-2 referring to IRCCS S. Orsola hospital treated with an early antiviral therapy were enrolled. A comparison between patients treated with nirmatrelvir/ritonavir (NTV/r), molnupiravir (MPV) and remdesivir (RDV) was conducted in term of indications and outcome. To account for differences between treatment groups a propensity score analysis was performed. After estimating the weights, we fitted a survey-weighted Cox regression model with inverse-probability weighting with hospital admission/death versus clinical recovery as the primary outcome.

**Results:**

Overall 1342 patients were enrolled, 775 (57.8%), 360 (26.8%) and 207 (15.4%) in MPV, NTV/r and RDV group, respectively. Median age was 73 (59–82) years, male sex was 53.4%. Primary indication was immunosuppression (438, 32.6%), the median time from symptom onset to drug administration was 3 [2–4] days. Overall, clinical recovery was reached in 96.9% of patients, with hospital admission rate of 2.6%. No significant differences were found in clinical recovery nor hospitalization. Cox regression showed a decreased probability of hospital admission/ death among prior vaccinated patients compared with unvaccinated (HR 0.31 [95%CI 0.14–0.70], p = 0.005]). No difference in hospitalization rates in early treatment compared to late treatment were found.

**Conclusions:**

No differences among MPV, NTV/r and RDV in terms of clinical recovery or hospitalization were found. Patients not vaccinated had a significant increased risk of hospitalization.

**Supplementary Information:**

The online version contains supplementary material available at 10.1186/s12879-023-08538-9.

## Introduction

Since the first outbreak of Severe Acute Respiratory Syndrome Coronavirus-2 (SARS-CoV-2) infection in late 2019 in Wuhan, and the subsequent pandemic, the natural history of Coronavirus disease-19 (COVID-19) significantly changed, due to the emerge of new variants, the baseline immunological status, the impact of the vaccination campaign and – not lastly – the development of new drugs with specific activity against SARS-CoV-2 [1, 2]. Initially, three different antivirals – namely nirmatrelvir/ritonavir (NTV/r), molnupiravir (MPV) and remdesivir (RDV) - were available for early treatment demonstrating a significant reduction in hospitalization due to disease worsening. Subsequently, MPV has been withdrawn in Europe and other new antivirals are in investigation. Although initially RDV was administered in patient with radiological evidence of pneumonia, it has recently shown a net benefit as early treatment in mild disease, demonstrating a 87% lower risk of hospitalization compared to placebo [3]. However, such drug has the limitation of the intravenous route of administration. Thereafter, two new oral antiviral drugs has been licensed with encouraging results, NTV/r and MPV, showing a reduction in hospitalization rate and related death [4, 5]. However, all these randomized trials have several limitations. First of all, recruited patients were unvaccinated, and secondly omicron was not the predominant variant at enrollment. In addition, the rate of immunosuppressed patients was very low. Considering the high percentage of vaccinated patients, classical endpoints adopted in registration trials (i.e. hospitalization rate, mortality) are outdated and not able to define the real clinical impact of early antiviral treatment. To date, large real-life studies conducted among Omicron surge demonstrated a decrease in hospital admission in patients treated with NTV/r compared with no treatment [6, 7]. Although MPV is no longer recommended in European countries, another large real-life study confirmed a favorable impact on COVID-related mortality even for such drug [8]. In addition, hospitalization rates of patients treated with MPV were similar to those treated with NTV/r, even if this latter group had lower underlying high-risk conditions [9].

However, real-life studies comparing the efficacy of both RDV, MPV and NTV/r and their impact on symptoms resolutions and viral clearance are limited. Based on these assumptions, we conducted a real-life study enrolling patients treated with early antiviral treatment during omicron surge.

## Materials and methods

### Study design & setting

This retrospective observational study was carried out from January 2022 until October 2022 at Policlinico di S. Orsola, a Tertiary-University Hospital in Bologna, in Northern Italy. All adult outpatients tested positive for SARS-CoV-2 referring to our center eligible for an early antiviral treatment, namely NTV/r, MPV or RDV, were enrolled. COVID-19 severity was assessed according to current guidelines [10]. Mild disease was considered as the presence of any of the various signs and symptoms of COVID-19 but do not have shortness of breath, dyspnea, or abnormal chest imaging. Moderate disease was defined as evidence of lower respiratory disease during clinical assessment or imaging and who have an oxygen saturation measured by pulse oximetry (SpO2) ≥ 94% on room air at sea level. All antiviral treatments during the study period were available. Diagnosis of SARS-CoV-2 infection was made through a polymerase chain reaction (PCR)-based or antigen test, patients with a previous infection and/or a previous antiviral treatment within 90 days from the diagnosis were excluded. The eligibility for antiviral treatment was dictated by the indications provided by the Italian Drug Agency (AIFA, agenzia italiana del farmaco) considering the presence of risk factors for disease progression along with the time from symptoms onset. Specifically, eligibility criteria were the same for each antiviral and included: i) the clinical evidence of a mild disease not requiring hospitalization, ii) ≤ 5 days from symptoms onset or positive test (whichever occurred first) and drug administration for NTV/r or MPV and ≤ 7 days for RDV, iii) the presence of at least a risk factor. Risk factors for disease were common between antivirals: age ≥ 65 years, primary or secondary immunodeficiency, severe cardiac diseases, severe pulmonary diseases, chronic liver diseases, severe neurological disorders, body mass index ≥ 30, uncontrolled diabetes and chronic renal failure. Asymptomatic patients were not included according to AIFA indications, as well as patients needing hospitalization at the time of evaluation. Patients with symptoms attributable to COVID-19 admitted to emergency department were systematically evaluated by an infectious disease (ID) physician. If the patient met the eligibility criteria, an antiviral drug was proposed. Furthermore, a day-hospital for both oral and infusive treatment administration, managed by both ID physicians and nurses staff during the entire study period was available. In addition, primary care physicians or other physicians outside the hospital could contact our service and propose symptomatic SARS-CoV-2 positive patients for an early treatment. The choice of a specific antiviral was evaluated by the infectious disease physician following principles of good clinical practice and not dictated by the study protocol. In particular, NTV/r was administered in absence of significative drug-drug interactions, at 300/100 mg twice daily or reduced to 150/100 mg twice daily in patients with an Estimated Glomerular Filtration Rate (eGRF) between 30 and 60 mL/min for 5 days. MPV was administered at 800 mg twice daily for 5 days regardless of renal clearance. Finally, RDV was administered with a loading dose of 200 mg at day one, followed by a single dose of 100 mg for two days, in absence of severe liver impairment or an end-stage renal disease.

### Patient consent statement

The study was approved by the Ethic Committee of the promoting center (CE-AVEC, Comitato Etico Indipendente di Area Vasta Emilia Centro, n. 283/2020/Oss/AOUBo, EM 242–2021). The need for written informed consent was waived by the CE-AVEC (Comitato Etico Indipendente di Area Vasta Emilia Centro) ethics committee due to retrospective observational nature of the study. All methods were performed in accordance with the relevant guidelines and regulations in accordance with the Declaration of Helsinki.

### Study definitions & endpoint variables

Demographics data (age, sex and comorbidities), as well as the date of symptoms onset/positive nasopharyngeal swab and last vaccination dose (when administered) were collected. The choice of the antiviral and the eligibility criteria were recorded and not dictated by study protocol. Patients were daily evaluated during antiviral treatment and followed until 30-day from diagnosis through phone call in order to evaluate the onset of drug-related adverse events according with common toxic criteria definition. The primary outcome was clinical recovery defined as resolution of symptoms, hospital admission due to COVID-19 progression and/or death whichever occurred first during follow-up period. Secondary endpoints included virological clearance, and timing to symptoms resolution.

### Statistical analysis

Continuous variables were expressed as median and interquartile range (IQR) and compared using Kruskall-Wallis test for comparisons between three drugs. The assumption of normality of the variables was tested through the skewness and kurtosis test for normality as well as visual inspections. Categorical variables were reported as counts and percentages and compared with Pearson’s chi-squared test or Fisher’s exact test, as appropriate.

To investigate protective and risk factors for hospital admission or death versus clinal recovery, univariable Cox regression analyses were performed. Hazard ratios (HRs) and their corresponding 95% confidence intervals (95% CIs) were estimated.

**Fig. 1:**
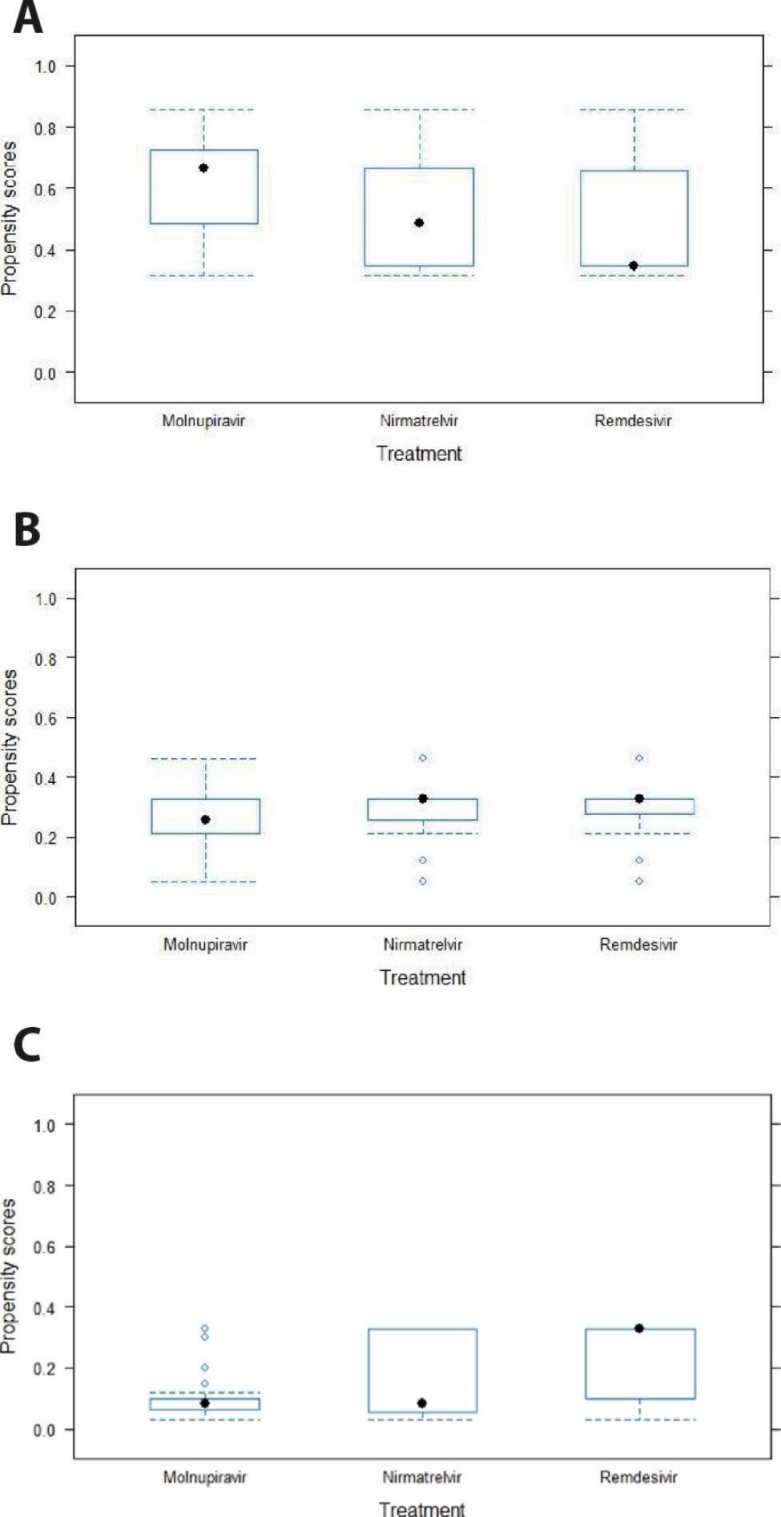
Boxplot by treatment group of the estimated propensity scores. **a**. Molnupiravir propensity scores by treatment group **b**. Nirmatrelvir propensity scores by treatment group **c**. Remdesivir propensity scores by treatment group

To control for imbalances between three drug treatments (i.e. NTV/r, MPV, RDV) on covariates included in indication we used a propensity score technique using inverse probability of treatment weighting as implemented by package twang (Toolkit for Weighting and Analysis of Nonequivalent Groups) in R [11]. This technique estimates weighting for each treatment using generalized boosted models and performing multinomial propensity scores [12]. When comparing three alternative treatments, the causal effect of interest was Average Treatment Effect (ATE), while the mean of Absolute Standardized Mean Difference (ASMD also referred to as the absolute standardized bias or effect size [ES]) was chosen as stopping rule. A key assumption in propensity score analyses is that each patient has a non-zero probability of receiving each treatment. The plausibility of this assumption may be assessed by examining the overlap of the empirical propensity score distributions (see Fig. 1). The assessment of balance between these three drug treatments on each covariate included in indication is reported in **Suppl. Figures 1–4** and **Suppl. Table 1** as well.

After estimating the weights, we fitted a survey-weighted Cox regression model with inverse-probability weighting [13] with hospital admission or death versus clinical recovery as the primary outcome adjusted for sex, vaccination status, COVID-19 severity, and time from symptoms onset to drug administration (expressed as days) as time-varying covariate. Visual inspection of survival curves for each covariate included in the Cox model were performed and time from symptoms onset to drug treatment was then included as time-varying covariate (data not shown). All the analyses were carried out using Stata 16.1 (Stata Corp., College Station, TX, USA) and R 4.2.1 (R Core Team, R Foundation for Statistical Computing, Vienna, 2022).

## Results

Overall, 1342 patients were enrolled during the study period. MPV was the antiviral more frequently prescribed (775, 57.8%) followed by NTV/r (360, 26.8%) and RDV (207, 15.4%). Characteristics of the study population is shown in Table 1. The median age was 73 (59–82) years, more than half of patients (716, 53.4%) were males. The primary indication for antiviral prescription was immunosuppression, both primary or acquired (438, 32.6%). Specifically, hematological malignancies, solid organ transplantation, active solid tumor, and hematological stem cell transplantation accounted for 40.0%, 28.1%, 23.6% and 1.8%, respectively. Briefly, other indications for drug prescriptions were severe cardiac disease (266, 19.8%), age ≥ 65 years (194, 14.5%) and chronic respiratory diseases (136, 10.1%). Almost all patients had a mild disease at presentation (1306, 98.6%), with a median time from symptoms onset to antiviral treatment of 3 (IQR 2–4) days. The majority of the study population received a prior SARS-CoV-2 vaccination (1247, 94.1%), in 78.9% of cases with a booster dose, within a median time of 144 (IQR 101–193) days prior to antiviral administration. Overall, 1269 (97.9%) patients reached negative nasopharyngeal swab within 8 (IQR 6–12) days from antiviral prescription, as well as clinical recovery was observed in 96.9% of cases within a median of 7 (IQR 6–11) days. Accordingly, hospital admissions due to worsening of the disease and COVID-19-related deaths occurred in 34 (2.6%) and 7 (0.5%) patients, respectively. Patients with unfavorable outcome mainly had end-stage chronic pulmonary diseases or kidney injury. Finally, 88 (7.9%) patients reported an adverse event within 30 days from antiviral treatment administration, none of such cases were classified as severe or required hospitalization.

**Table 1:** Descriptive analysis among non-complicated COVID-19 patients treated with early antiviral treatment

	Molnupiravir (N = 775, 57.8%)	Nirmatrelvir/r (N = 360, 26.8%)	Remdesivir (N = 207, 15.4%)	Overall (N = 1342)	p-value
Demographic data					
Age (years), median (IQR)	78 (66–84)	67 (53–76)	64 (49–74)	73 (59–82)	< 0.001
Sex, male	417 (53.9)	180 (50.0)	119 (57.2)	716 (53.4)	0.228
**Indication**					
BMI ≥ 30	44 (5.7)	42 (11.7)	6 (2.9)	92 (6.9)	< 0.001
Chronic respiratory diseases	90 (11.6)	35 (9.7)	11 (5.3)	136 (10.1)	0.026
Uncontrolled diabetes	21 (2.7)	7 (1.9)	3 (1.4)	31 (2.3)	0.565
Chronic hepatic diseases	2 (0.3)	0 (0.0)	1 (0.5)	3 (0.2)	0.541
Age ≥ 65 years	126 (16.3)	61 (16.9)	7 (3.4)	194 (14.5)	< 0.001
Immunosuppression	152 (19.6)	143 (39.7)	143 (68.8)	438 (32.6)	< 0.001
SOT	45 (38.1)	1 (1.0)	48 (39.7)	94 (28.1)	
Solid tumour	41 (34.8)	21 (21.9)	17 (14.1)	79 (23.6)	
Hematologic malignancy	22 (18.6)	63 (65.6)	49 (40.5)	134 (40.0)	
HSCT	0 (0.0)	4 (4.2)	2 (1.7)	6 (1.8)	
Primitive immunodeficiency	4 (3.4)	4 (4.2)	2 (1.7)	10 (3.0)	
Chronic renal failure	113 (14.6)	7 (1.9)	12 (5.8)	132 (9.8)	< 0.001
Haemodialysis	27 (3.5)	0 (0.0)	0 (0.0)	27 (2.0)	
Cardiovascular diseases	193 (24.9)	56 (15.6)	17 (8.2)	266 (19.8)	< 0.001
Neurodegenerative diseases	13 (1.7)	7 (1.9)	5 (2.4)	25 (1.9)	0.719
**COVID-19 severity**					
Mild	758 (98.7)	354 (98.6)	194 (98.0)	1306 (98.6)	0.719
Moderate	10 (1.3)	5 (1.4)	4 (2.0)	19 (1.4)	
Time from symptoms onset to treatment (days), median (IQR)	3 (2–3)	2 (2–3)	3 (2–5)	3 (2–4)	< 0.001
Time from symptoms onset to treatment (days)					
0–1	137 (17.9)	78 (22.0)	20 (10.9)	235 (18.1)	< 0.001
2	238 (31.2)	100 (28.3)	41 (22.3)	379 (29.1)	
3	209 (27.4)	95 (26.8)	44 (23.9)	348 (26.7)	
≥ 4	180 (23.6)	81 (22.9)	79 (42.9)	340 (26.1)	
**Vaccination**					
Not vaccinated	33 (4.3)	24 (6.7)	23 (11.6)	80 (6.0)	< 0.001
Vaccinated	737 (95.8)	334 (93.0)	176 (89.3)	1247 (94.1)	
3 doses	567 (77.3)	284 (84.8)	131 (74.4)	982 (78.9)	
Time to last dose to diagnosis (days), median (IQR)	145 (100–193)	142 (105–189)	146 (101–208)	144 (101–193)	0.636
**Events**					
Clinical recovery (CR)	724 (96.3)	344 (97.7)	198 (97.5)	1266 (96.9)	0.633
Hospitalization	23 (3.1)	6 (1.7)	5 (2.5)	34 (2.6)	
Death	5 (0.8)	2 (0.6)	0 (0.0)	7 (0.5)	
Time from treatment start to CR (days), median (IQR)	8 (6–12)	7 (6–9)	8 (5–12)	7 (6–11)	< 0.001
Virological recovery (VR)	730 (98.1)	338 (96.6)	201 (99.5)	1269 (97.9)	0.065
Time from treatment start to VR (days), median (IQR)	8 (6–13)	7 (6–9)	8 (6–13)	8 (6–12)	< 0.001
Mild adverse events	45 (6.9)	37 (12.3)	6 (3.9)	88 (7.9)	0.003

**Abbreviations**: BMI, body mass index; SOT, solid organ transplantation; HSCT, haematopoietic stem cell transplantation

All values given are n (%) unless otherwise stated

A comparison among the three classes of antivirals is shown in Table 1. Patients treated with MPV were on average older (median 78 years, p < 0.001), affected by chronic renal failure (14.6%, p < 0.001) and with severe cardiac conditions (24.9%, p < 0.001). NTV/r was prescribed more frequently in obese patients (11.7%, p < 0.001), and earlier compared to other treatments (2 days from symptoms onset, p < 0.001). In addition, patients treated with NTV/r showed a significant impact on time reduction for both virological and clinical recovery (median 7 days, < 0.001). However, a significant increased rate of mild adverse events was reported (12.3%, p = 0.003). All adverse events were classified as grade 1 and in any case drug discontinuation was necessary. Finally, patients treated with RDV were significantly younger (median 64 years, p < 0.001) but more frequently immunocompromised (69.1%, p < 0.001) and unvaccinated (11.2%, p = 0.002) compared with the other groups. Despite such heterogeneous population, no difference in term of clinical recovery rates, virological recovery rates and rates of hospitalization were observed.

Thereafter, the univariable analysis for hospital admission or death was performed (Table 2). Patients with chronic respiratory diseases had an increased risk of hospitalization (HR 2.93 [95%CI 1.46–5.90], p = 0.003). Compared to MPV, a trend toward better outcomes for NTV/r (HR 0.70 [0.32–1.53], p = 0.368) and RDV (HR 0.59 [0.23–1.55], p = 0.283) was observed. A delay in antiviral treatment does not seem to affect rates of clinical recovery (HR 0.85 [0.67–1.09] for each day, p = 0.207), whereas prior vaccination was protective (HR 0.38 [0.15–0.98], p = 0.045).

**Table 2:** Univariable analysis for hospital admission or death among non-complicated COVID-19 patients treated with early antiviral treatment

	HR (95% CI)	p-value
Demographic data		
Age (years)*	1.03 (1.01–1.06)	0.007
Sex (male)	0.93 (0.50–1.73)	0.813
**Indication**		
BMI ≥ 30	0.42 (0.06–3.04)	0.387
Chronic respiratory diseases	2.93 (1.46–5.90)	0.003
Uncontrolled diabetes	1.25 (0.17–9.12)	0.825
Chronic hepatic diseases	-	-
Age ≥ 65 years	1.19 (0.50–2.83)	0.701
Immunosuppression	0.87 (0.45–1.69)	0.681
Chronic renal failure	1.22 (0.48–3.12)	0.677
Cardiovascular diseases	0.44 (0.16–1.24)	0.122
Neurodegenerative diseases	-	-
Drug		
Molnupiravir	Reference	
Nirmatrelvir	0.70 (0.32–1.53)	0.368
Remdesivir	0.59 (0.23–1.55)	0.283
**COVID-19 severity**		
Mild	Reference	
Moderate	10.15 (4.04–25.50)	< 0.001
Time from symptoms onset to treatment (days)*	0.85 (0.67–1.09)	0.207
**Vaccination**		
Vaccinated	0.38 (0.15–0.98)	0.045
Time to last dose to diagnosis (days)*	0.99 (0.99–1.01)	0.801
**Events**		
Time from treatment start to VR (days)*	1.06 (1.03–1.09)	< 0.001

h: Hazard Ratio

*For each year or day

The overlap of the empirical propensity score distributions shown in Fig. 1 proves that the assumption of non-zero probability of receiving each treatment seems to be met. Of note, Suppl Figs. 1–4 along with Suppl Table 1 supported that an optimal balance was reached as well.

**Fig. 2:**
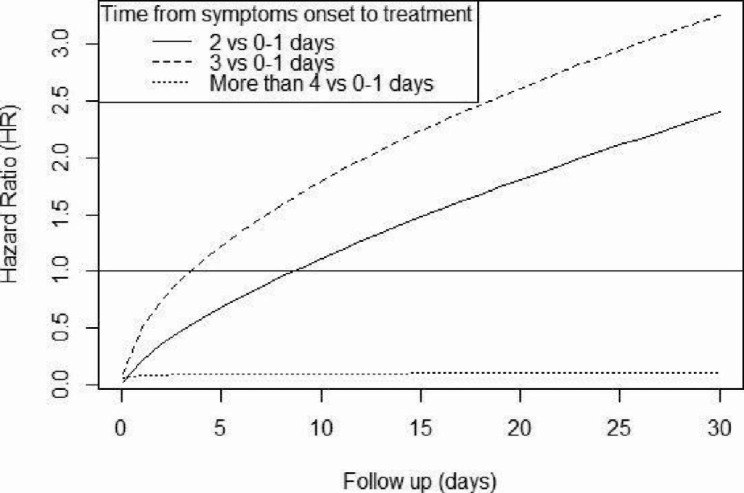
Plot of time-varying HR of hospital admission or death for time from symptoms onset to treatment as per survey-weighted Cox model. Reference category: 0–1 days. Curves were estimated using the formula reported in the notes of Table 3

As expected, at multivariable analysis prior vaccination was independently associated with clinical recovery (HR 0.31 [95%CI 0.14–0.70], p = 0.005). Conversely, a moderate disease has been associated with hospitalization (HR 12.23 [4.65–32.18], p < 0.001) (Table 3). The plot of time-varying HR for time from symptoms onset to drug administration is reported in Fig. 2. We found a positive effect for patients treated from the fourth day onwards. This might be related to low rate of hospitalization (2.6%) and unbalanced distribution of hospitalization/death and clinical recovery by follow-up time (**Suppl. Table 2**).

**Table 3:** Multivariable Cox model for hospital admission or death among non-complicated COVID-19 patients treated with early antiviral treatment

	HR_adj_ (95% CI)^a^	p-value
Vaccination		
No	Reference	
Yes	0.31 (0.14–0.70)	0.005
**Covid-19 severity**		
Mild	Reference	
Moderate	12.23 (4.65–32.18)	< 0.001
**Time from symptoms onset to treatment (days)** ^**b**^ **(with time-varying HR)** ^**c**^		
0–1	Reference	
2	0.22 (0.05–1.11)	0.068
3	0.51 (0.04-6.00)	0.593
More than 4	0.09 (0.01–0.66)	0.019

^a^Survey-weighted Cox regression model with inverse-probability weighting and design-based standard errors. HR_adj_: Hazard Ratio adjusted for sex

^b^Categories are based on quartiles

^c^Time-varying HR (HR_tv_) was calculated using: HR_tv_=exp [β_1_X + β_2_Xlog(t)] = HR(X)*exp[β_2_Xlog(t)]. For each category of time from symtpoms onset to treatment, we considered:

-category “2 days”: β_1_= -1.50, β_2_ = 0.70, HR = 0.22

HR_tv_=exp[-1.50 + 0.70*log(t)] = 0.22*exp[0.70*log(t)]

-category “3 days”: β_1_= -0.67, β_2_ = 0.54, HR = 0.51

HR_tv_=exp[-0.67 + 0.54*log(t)] = 0.51*exp[0.54*log(t)]

-category “More than 4 days”: β_1_= -1.18, β_2_ = 0.08, HR = 0.09

HR_tv_=exp[-1.18 + 0.08*log(t)] = 0.09*exp[0.08*log(t)]

The plot of these HR_tv_ is reported in Fig. 2

## Discussion

For our knowledge, this is the first real-world study focused on a large cohort of outpatients affected by non-severe COVID-19 but with high risk of progression, comparing the efficacy of RDV, MPV and NTV/r as early antiviral treatment. No differences in term of hospitalization rate were found among the different cohorts. However, prior vaccination seems to represent the driving force to favorable outcome. Within the window period of 5–7 days in which antivirals could be prescribed, we did not find a correlation between tempestive treatment and favorable outcome.

Clinical trials evaluating the effectiveness of antiviral drugs as early treatment showed a net benefit in term of hospitalization and disease progression compared to placebo, but were conducted enrolling unvaccinated patients during previous variants [5, 14]. These characteristics are outdated, considering the omicron surge and the vaccination campaign. Since these variables had a meaningful impact on the natural history of COVID-19, hospitalization rates and related deaths are probably no more reliable endpoints able to establish the real efficacy of early antiviral treatment. In fact, a recent comparison study demonstrated a lower risk of severe outcome following SARS-CoV-2 infection for omicron compared to delta due to a lower intrinsic severity of the variant [15]. The risk of disease progression was higher in unvaccinated individuals, but it remains lower in omicron variant if compared to delta. Latest studies showed a risk of hospitalization due to progression of the disease during the omicron surge attesting up to 5%, but such results are not adjusted for antiviral prescription or individual risk factors [16, 17].

As reported by an extremely wide English cohort of eligible patients, the use of antiviral treatments increased over time, from 9 to 29%. However, hospitalization rate in untreated patients was quite low, attesting at 2% [18]. In our cohort, the overall hospitalization rate was 2.6%. These relatively low rates of hospital admission could be explained considering a wide percentage of vaccinated patients (94.1%), despite a high prevalence of immunosuppressed patients and an elderly population.

Real world data comparing patients treated with new oral antivirals or sotrovimab have been published. A study found that sotrovimab was associated with a lower risk of severe outcome than molnupiravir [19]. Another study reported similar efficacy of sotrovimab, NTV/r and MPV in preventing severe outcomes [20]. However, an in vitro decrease of efficacy in new subvariants for sotrovimab has been found, raising concerns about its current place in therapy.

The efficacy of NTV/r and MPV was evaluated in a large cohort of patients hospitalized with a mild disease during the omicron BA.2 surge [8]. Both drugs demonstrated a net benefit compared with controls. Another retrospective study performed a cost-analysis of such drugs both in out- and inpatients, confirming previous results [21]. Recently, a real-life study comparing NTV/r to MPV in outpatients was performed [22]. One-hundred forty-six in the MPV group and 111 patients in the NTV/r were enrolled, with a hospitalization rate of 0.9% and 2.1%, respectively, within 14-day follow-up. Of interest, the authors found a median time to negative nasal swab significantly lower for NTV/r (8 vs. 10 days). Another recent study confirmed a decreased viral shedding in patients treated with NTV/r over MPV or sotrovimab [20]. Similarly, in our cohort patients treated with NTV/r showed an early negativity of nasal swab compared to other antivirals (7 vs. 8 days). Such findings could be useful in patients awaiting invasive procedures or immunosuppressive treatments in which a negative test is mandatory. Along with that, NTV/r was associated with a reduced time of disease related symptoms compared to MPV and RDV treatment. Nevertheless, it is important to note that patients in the other groups were on average older and with a lower rate of prior vaccination. NTV/r was the drug with the highest rate of adverse events, attesting at 12.3%. These results are consistent with previous studies [14]. Additionally, a recent meta-analysis showed a similar risk of any adverse events comparing antiviral agents to placebo [23]. Of note, in our cohort none of the adverse events needed a medical intervention or the discontinuation of the drug.

The RDV cohort had probably the highest risk of progression, considering that almost 70% of treated patients were immunosuppressed and 11.1% of these were unvaccinated. Despite such characteristics, the overall hospitalization rate in this subgroup was 2.6%, similar to other cohorts with a high prevalence of vaccinated individuals [24]. However, patients treated with RDV could represent a selected population due to the need of a dedicated service composed by both medical and nursing staff.

Patients treated with MPV were on average elderly (median age 78 years) and more likely affected by end stage renal disease compared to other groups. However, in this subgroup the rate of hospitalization was 2.6%. Despite a younger median age, in a cohort of 145 fully vaccinated outpatients treated with MPV rates of hospital admission were similar, attesting at 2.7% [25]. Different real-life studies compared the efficacy of MPV and NTV/r in high-risk outpatients, founding similar results for both early antiviral treatments [9, 26], pointing out that the choice of a specific antiviral drug over another is not the main variable able to influence the outcome of the patient. De Vito et al. published a cohort of 192 patients with a mild disease treated with MPV [27], showing a progression rate of 10.4% despite a younger population (median 70 years). Such results could be influenced by a relatively low rate of previous vaccination (56.8%), suggesting that vaccination status is one of the most important factors influencing disease progression. In fact, several studies highlighted the role of vaccination in preventing the worsening of the disease, considering that unvaccinated people has a 10-fold risk of hospitalization during omicron surge [28]. Accordingly, in our study, vaccinated individuals had more than a 3-fold probability of recovery compared to unvaccinated patients.

In contrast to what has been observed with influenza infection [29], in which an early antiviral initiation reduces the likelihood of severe outcomes, a tempestive approach seems not to have a clinical impact on patients with mild COVID-19 infection at high risk of progression. Timeline for treatment administration derives from clinical trials results, for instance 5 days for oral drugs and 7 days to remdesivir from symptoms onset. Thus, it is reasonable to think that favorable outcome is related to a tempestive approach. However, in a high prevalence of vaccinated patients this assumption seems not to be confirmed. In fact, we did not notice any difference in term of outcomes when compared early (0–1 days) prescriptions with late (4–5 days) prescriptions, neither in immunosuppressed patients. This latter observation suggests that antiviral prescription may not be seen as an emergency treatment, but it should be carefully tailored by the attending physician, considering pros and cons for each available drug.

Considering the observational nature of the study, it has intrinsic limitations. First of all, an allocation bias could occur among the three cohorts. In addition, we did not perform a systematic identification of variants, but epidemiological studies conducted in Italy during the study period confirmed omicron as the predominant circulating variant. In addition, each drug has specific contra-indications that may limit the prescription (i.e. severe chronic kidney injury for NTV/r and RDV or major drug-drug interactions for NTV/r). Intravenous route is mandatory for RDV and such issue could have reduced RDV prescription, however we created a specific service managed by ID physicians and nurses to increase the availability of this treatment, whenever suggested. Moreover, we believe that our cohort and subsequent results could be consistent with real-life populations, helping clinicians in antiviral choice, as these limitations are commonly experienced in real-life. In addition, the use of a propensity score analysis could have reduced selection bias. Finally, the rates of clinical rebounds, especially with NTV/r, as well as the rates of persisting COVID were not collected.

In conclusion, we do not find significant differences in terms of clinical recovery among NTV/r, MPV and RDV treatment, while vaccination status has a key role on disease progression and subsequent hospitalization. NTV/r was associated with both lower time to negative nasal swab as well as faster clinical recovery, along with a higher rate of mild adverse events. Thereby the main driver of the drug choice should be represented by a careful assessment of the patient clinical condition with particular attention to drug history, avoiding a decision-making based primarily on antiviral activity and taking into account the patient related risk of adverse events or drug-drug interactions.

### Electronic supplementary material

Below is the link to the electronic supplementary material.

Supplementary Material 1

Supplementary Material 2

Supplementary Material 3

Supplementary Material 4

Supplementary Material 5

Supplementary Material 6

## Data Availability

The datasets used and/or analysed during the current study are available from the corresponding author on reasonable request.
