# Supplementary material for: Comparison between available early antiviral treatments in outpatients with SARS-CoV-2 infection: a real-life study
Source: BMC Infect Dis. 2023 Oct 2;23:646. doi: 10.1186/s12879-023-08538-9 (PMC10546723; doi:10.1186/s12879-023-08538-9)
Supplement: Supplementary file 1 — Supplementary Material 1 [file 12879_2023_8538_MOESM1_ESM.docx]

**Supplementary Table 1.** Percentage for treatment groups for each covariate included in indication for treatment before and after weighting.

|  |  |  | **Unweighted** | | | | **Weighted (ES.mean)** | | | |
| --- | --- | --- | --- | --- | --- | --- | --- | --- | --- | --- |
| **Treatment 1** | **Treatment 2** | **Pre-treatment variable** | **%1** | **%2** | **std.eff.sz** | **p** | **%1** | **%2** | **std.eff.sz** | **p** |
| Molnupiravir | Nirmatrelvir | BMI≥30 | 5.7 | 11.7 | 0.238* | 0.000 | 6.8 | 6.8 | 0.003 | 0.961 |
| Molnupiravir | Nirmatrelvir | BPCO | 11.6 | 9.7 | 0.063 | 0.344 | 10.2 | 10.2 | 0.001 | 0.982 |
| Molnupiravir | Nirmatrelvir | Diabete | 2.7 | 1.9 | 0.051 | 0.44 | 2.3 | 2.3 | 0.002 | 0.980 |
| Molnupiravir | Nirmatrelvir | Hepatic disease | 0.3 | 0.0 | 0.055 | 0.335 | 0.2 | 0.0 | 0.045 | 0.158 |
| Molnupiravir | Nirmatrelvir | Age≥30 years | 16.4 | 16.9 | 0.016 | 0.814 | 14.5 | 14.6 | 0.001 | 0.989 |
| Molnupiravir | Nirmatrelvir | Immunosuppression | 19.6 | 39.7 | 0.429* | 0.000 | 32.6 | 32.7 | 0.001 | 0.991 |
| Molnupiravir | Nirmatrelvir | Chronic renal failure | 14.6 | 1.9 | 0.424* | 0.000 | 9.8 | 10.0 | 0.006 | 0.960 |
| Molnupiravir | Nirmatrelvir | Cardiovascular disease | 24.9 | 15.6 | 0.234* | 0.000 | 19.8 | 19.9 | 0.002 | 0.973 |
| Molnupiravir | Nirmatrelvir | Neurodegenerative disease | 1.7 | 1.9 | 0.020 | 0.75 | 1.9 | 1.9 | 0.002 | 0.981 |
| Molnupiravir | Remdesivir | BMI≥30 | 5.7 | 2.4 | 0.130 | 0.056 | 6.8 | 6.8 | 0.003 | 0.983 |
| Molnupiravir | Remdesivir | BPCO | 11.6 | 5.3 | 0.209* | 0.008 | 10.2 | 10.1 | 0.002 | 0.982 |
| Molnupiravir | Remdesivir | Diabete | 2.7 | 1.4 | 0.084 | 0.297 | 2.3 | 2.3 | 0.001 | 0.995 |
| Molnupiravir | Remdesivir | Hepatic disease | 0.3 | 0.5 | 0.048 | 0.603 | 0.2 | 0.5 | 0.061 | 0.469 |
| Molnupiravir | Remdesivir | Age≥30 years | 16.4 | 3.4 | 0.369* | 0.000 | 14.5 | 14.5 | 0.001 | 0.994 |
| Molnupiravir | Remdesivir | Immunosuppression | 19.6 | 69.1 | 1.055* | 0.000 | 32.6 | 32.5 | 0.002 | 0.981 |
| Molnupiravir | Remdesivir | Chronic renal failure | 14.6 | 5.8 | 0.295* | 0.001 | 9.8 | 9.8 | 0.000 | 0.998 |
| Molnupiravir | Remdesivir | Cardiovascular disease | 24.9 | 8.2 | 0.419* | 0.000 | 19.8 | 19.8 | 0.000 | 0.998 |
| Molnupiravir | Remdesivir | Neurodegenerative disease | 1.7 | 2.4 | 0.055 | 0.482 | 1.9 | 1.9 | 0.000 | 1.000 |
| Nirmatrelvir | Remdesivir | BMI≥30 | 11.7 | 2.4 | 0.368* | 0.000 | 6.8 | 6.8 | 0.000 | 0.998 |
| Nirmatrelvir | Remdesivir | BPCO | 9.7 | 5.3 | 0.146 | 0.065 | 10.2 | 10.1 | 0.001 | 0.994 |
| Nirmatrelvir | Remdesivir | Diabete | 1.9 | 1.4 | 0.033 | 0.667 | 2.3 | 2.3 | 0.002 | 0.983 |
| Nirmatrelvir | Remdesivir | Hepatic disease | 0.0 | 0.5 | 0.102 | 0.187 | 0.0 | 0.5 | 0.106 | 0.319 |
| Nirmatrelvir | Remdesivir | Age≥30 years | 16.9 | 3.4 | 0.385* | 0.000 | 14.6 | 14.5 | 0.002 | 0.989 |
| Nirmatrelvir | Remdesivir | Immunosuppression | 39.7 | 69.1 | 0.626* | 0.000 | 32.7 | 32.5 | 0.003 | 0.975 |
| Nirmatrelvir | Remdesivir | Chronic renal failure | 1.9 | 5.8 | 0.129* | 0.015 | 10.0 | 9.8 | 0.006 | 0.966 |
| Nirmatrelvir | Remdesivir | Cardiovascular disease | 15.6 | 8.2 | 0.184* | 0.012 | 19.9 | 19.8 | 0.002 | 0.987 |
| Nirmatrelvir | Remdesivir | Neurodegenerative disease | 1.9 | 2.4 | 0.035 | 0.708 | 1.9 | 1.9 | 0.002 | 0.985 |

*Effect size statistically significant

**Supplementary Table 2**. Distribution of hospitalization/death and clinical recovery by follow-up time.

|  | **Follow up (days)** | | | | | |
| --- | --- | --- | --- | --- | --- | --- |
|  | **0-5**  **n (%)** | **6-10**  **n (%)** | **11-15**  **n (%)** | **16-20**  **n (%)** | **21-25**  **n (%)** | **26-30**  **n (%)** |
| **Clinical recovery** | 260 (92.5) | 658 (98.2) | 223 (98.7) | 91 (97.8) | 26 (96.3) | 8 (80.0) |
| **Hospitalization/death** | 21 (7.5) | 12 (1.8) | 3 (1.3) | 2 (2.2) | 1 (3.7) | 2 (20.0) |
| **Total** | 281 | 670 | 226 | 93 | 27 | 10 |
