# Supplementary figures and images for: Comparison between available early antiviral treatments in outpatients with SARS-CoV-2 infection: a real-life study
Source: BMC Infect Dis. 2023 Oct 2;23:646. doi: 10.1186/s12879-023-08538-9 (PMC10546723; doi:10.1186/s12879-023-08538-9)

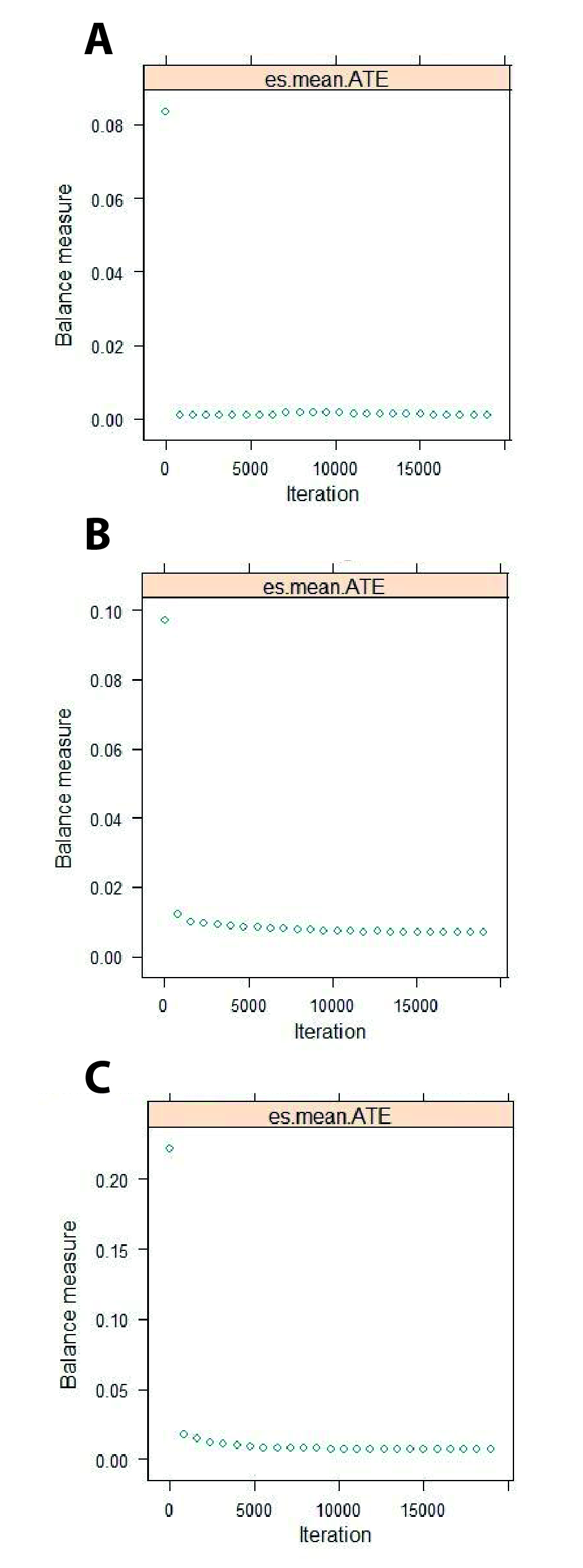

Supplement: Supplementary file 2 — Supplementary Material 2 [file 12879_2023_8538_MOESM2_ESM.jpg]

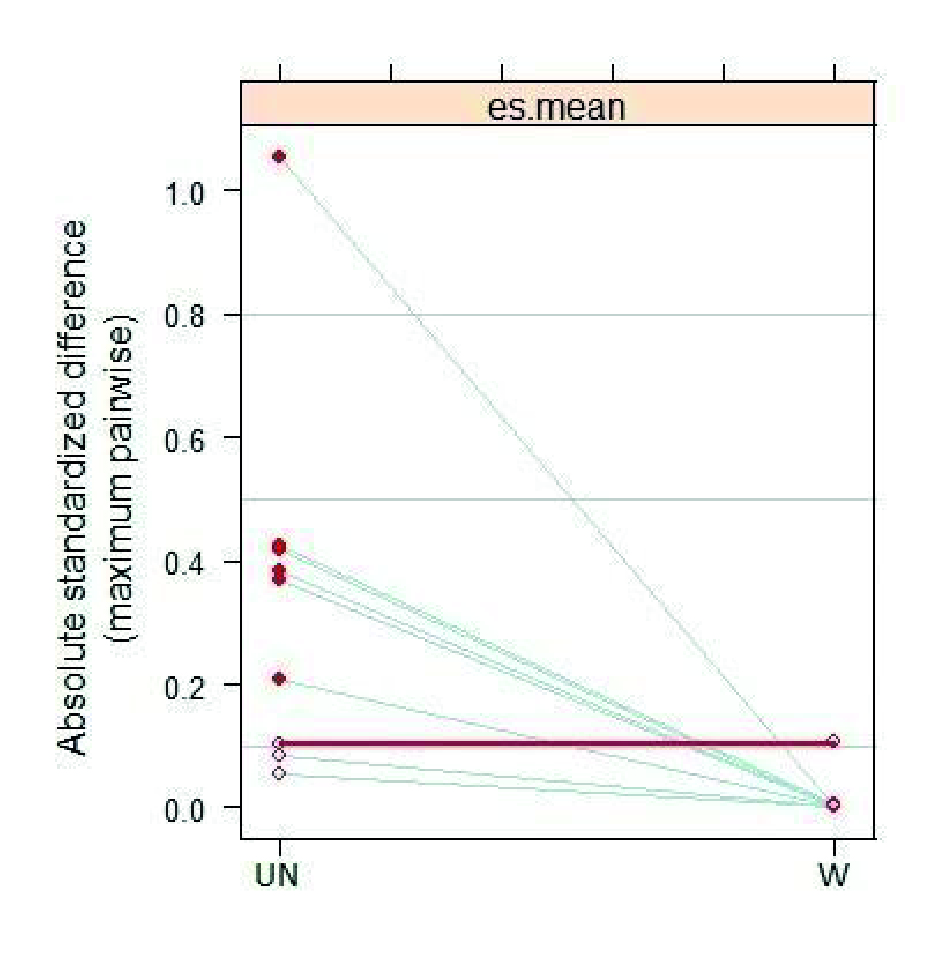

Supplement: Supplementary file 3 — Supplementary Material 3 [file 12879_2023_8538_MOESM3_ESM.jpg]

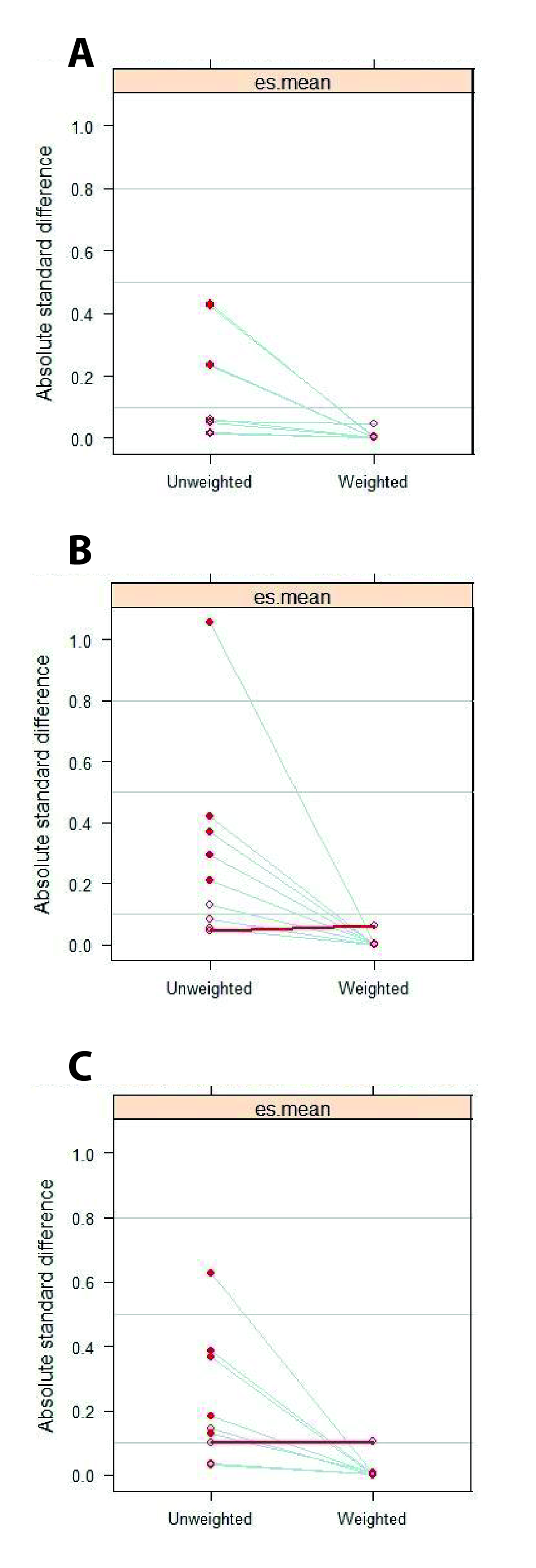

Supplement: Supplementary file 4 — Supplementary Material 4 [file 12879_2023_8538_MOESM4_ESM.jpg]

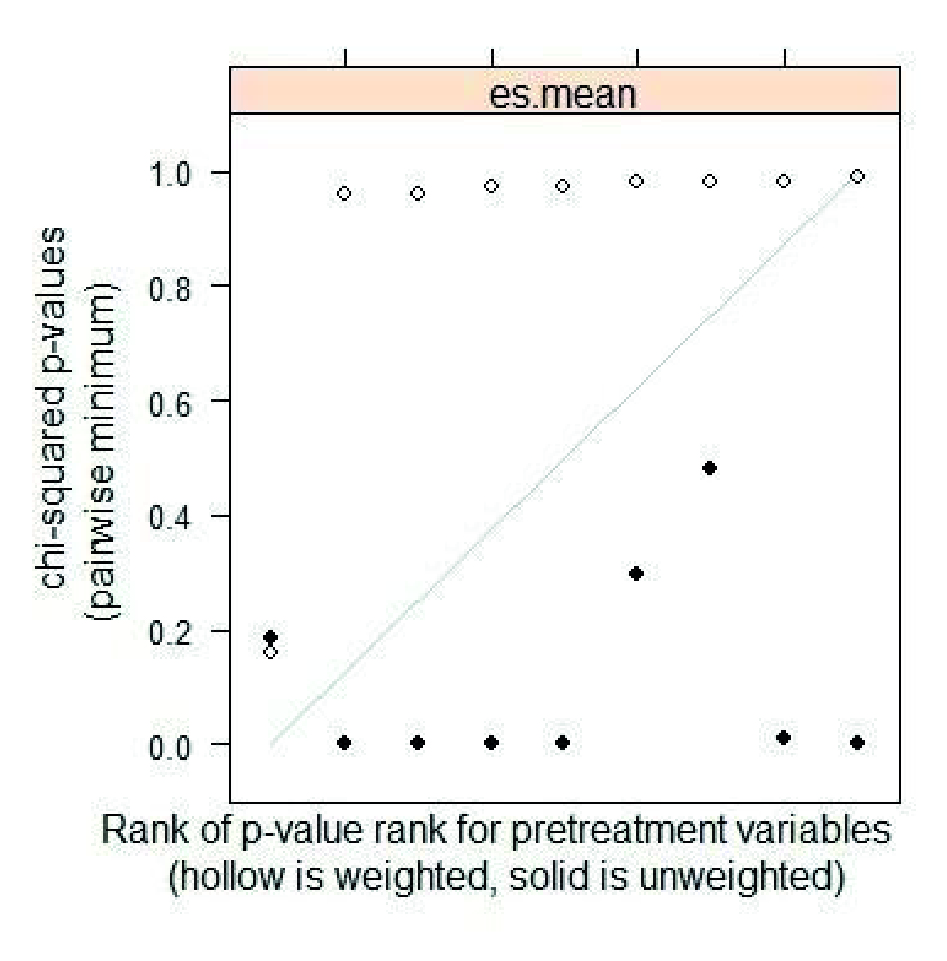

Supplement: Supplementary file 5 — Supplementary Material 5 [file 12879_2023_8538_MOESM5_ESM.jpg]
