# Supplementary material for: Comparison between available early antiviral treatments in outpatients with SARS-CoV-2 infection: a real-life study
Source: BMC Infect Dis. 2023 Oct 2;23:646. doi: 10.1186/s12879-023-08538-9 (PMC10546723; doi:10.1186/s12879-023-08538-9)
Supplement: Supplementary file 6 — Supplementary Material 6 [file 12879_2023_8538_MOESM6_ESM.docx]

**Supplementary Figure 1.** Number of iterations for each treatment against other two treatments.

a. Balance for Molnupiravir against others

b. Balance for Nirmatrelvir against others

c. Balance for Remdesivir against others

**Supplementary Figure 2.** Maximum pairwise AMSD (absolute mean standardized differences) for each covariate included in indication for treatment before and after weighting.

**Supplementary Figure 3.** AMSD (absolute mean standardized differences) for each comparison between two treatment groups before and after weighting.

a. Balance of Molnupiravir versus Nirmatrelvir

b. Balance of Molnupiravir versus Remdesivir

c. Balance of Nirmatrelvir versus Remdesivir

**Supplementary Figure 4.** P value for each covariate included in indication for treatment.
